# Human Trafficking Among North American Indigenous Women: A Scoping Review

**DOI:** 10.3390/nursrep16070246

**Published:** 2026-07-15

**Authors:** Deanna Thompson, Christine Hodgson, Timian M. Godfrey, Ruth E. Taylor-Piliae

**Affiliations:** 1 Great Falls College, Montana State University, Great Falls, MT 59405, USA; deanna.thompson@gfcmsu.edu; 2 School of Nursing, University of California San Francisco, San Francisco, CA 94143, USA; crhodgson@yahoo.com; 3 College of Nursing, The University of Arizona, Tucson, AZ 85721, USA; timiangodfrey@arizona.edu

**Keywords:** human trafficking, sex trafficking, sex exploitation, trafficking of women, Indigenous women

## Abstract

**Background/Objectives:** Human trafficking is the use of force, fraud, or coercion to compel a person to provide labor or sex and has become a public health crisis. This scoping review aimed to identify known risk factors for trafficking among North American Indigenous women and to synthesize the complexities of trafficking and its related health outcomes. **Methods:** Following the PRISMA-ScR checklist, a comprehensive literature search of seven electronic databases (CINAHL, Embase, PubMed, SCOPUS, PsycINFO, Cochrane Library, and Google Scholar) was conducted in March 2022 and updated in September 2023. English language research reports, literature reviews, dissertations and theses, commentaries, and government reports that focused on human trafficking in North American Indigenous women aged 18 years and older from the United States and Canada were identified using key terms. **Results:** Fifteen articles were included, revealing three themes: structurally produced vulnerabilities and other contributors to human trafficking, the long-term effects of human trafficking, and the need for collaboration of key stakeholders to mitigate human trafficking. Risk factors of human trafficking for Indigenous women include personal factors, historical trauma, and inadequate systems to identify and support victims. The needs of this population are complex. However, a multidisciplinary approach can be effective in combatting human trafficking. **Conclusions:** This review substantiates key risk factors and complexities unique to the trafficking experienced by North American Indigenous women, while also revealing a persistent gap in the research. These findings advance nursing knowledge by informing culturally responsive, trauma-informed care approaches while supporting targeted educational and research initiatives to improve health outcomes among these women.

## 1. Introduction

Human trafficking is defined internationally as the recruitment, transportation, transfer, harboring, or receipt of persons by means of force, fraud, or coercion for the purpose of exploitation, in accordance with Article 3 of the Protocol to Prevent, Suppress and Punish Trafficking in Persons, Especially Women and Children, supplementing the United Nations Convention against Transnational Organized Crime (Palermo Protocol) [1]. In Canada, trafficking in persons and exploitation are defined under sections 279.01 and 279.04 of the Criminal Code, and, where relevant to cross-border entry, under Section 118 of the Immigration and Refugee Protection Act [2]. In the United States, this definition is codified under the Victims of Trafficking and Violence Protection Act of 2000, Public Law 106-386 [3]. Human trafficking has become recognized as a public health crisis, thought to represent a 150-billion-dollar global industry and the second largest organized crime system [4], with over 700,000 persons—mainly women and children—trafficked internationally annually, and approximately 50,000 women and children trafficked into the United States annually [3,4]. The impact of human trafficking on the health of people who are victims (currently in a trafficking situation) and survivors (who have left the trafficking situation) has not been well studied [4]. Indigenous women are at a high risk of becoming victims of human trafficking, likely due to historical trauma, resulting contemporary social obstacles, and oppression [1,3,5]. An article from 2015 sought to determine the prevalence of human trafficking across four United States and Canadian cities where Indigenous women constitute less than 10% of the general population. A disproportionate 40% of women involved in sex trafficking identified as American Indian/Alaska Native (AI/AN) or First Nations. More research is needed about the health effects of human trafficking of women who identify as Indigenous First Peoples, Wendat, Anishinabek and Haudenosaunee of Canada, Mandan, Hidatsa, Arikara Nations, Northern Plains, Anishinaabe, Lakota, American Indian, and Alaska Native (hereafter referred to as North American Indigenous) [6,7].

There are multiple and complex reasons that Indigenous women are susceptible to human traffickers, including historical trauma, defined as, “cumulative emotional and psychological wounding across generations, including the lifespan, which emanates from massive group trauma” [8] (p. 288). The historical trauma response is a constellation of features found in some Indigenous people who have inherited or experienced massive group trauma, forced assimilation, colonization, and cultural losses. The historical trauma response can include psychological distress such as unresolved grief, depression, post-traumatic stress disorder (PTSD) and resulting substance use or other behavioral health issues [9]. The women and girls of Indigenous families who have experienced historical trauma or forced assimilation, low educational levels, substance use disorder, living in communities with economic disparity, or exposure to sexual and physical violence are at risk for human trafficking [6,7]. Recent Indigenous scholars have shifted the focus of historical trauma away from how an individual responds to intergenerational traumatic experiences and instead to addressing the dominant systems that cause the social and economic inequities of Indigenous groups [10].

While there has been an increased awareness of human trafficking, efforts to combat this activity have fallen short. Efforts have been hindered by disbelief that human trafficking occurs on domestic soil, and a failure to recognize and identify human trafficking victims who intersect with the healthcare system. As many as 88% of all victims reported having had an encounter with healthcare personnel during the time they were involved in trafficking [4]; however, many of those victims go unrecognized. Healthcare providers, particularly nurses working in emergency departments, urgent care settings, primary care clinics, gynecological clinics, community health centers, and pediatrics, are likely to encounter a person involved in human trafficking [11]. Nurses are uniquely positioned to identify and support trafficking victims, given their frequent, direct contact with a wide range of patients across these care settings, where a substantial proportion of trafficking victims have documented contact with healthcare providers [12]. However, victims often face barriers to disclosure, including shame, fear of judgment, and limited time with providers; trauma-informed, confidential approaches to care may help nurses create conditions more conducive to disclosure than other points of contact [13]. Given this access and potential for building trust, nurses represent key stakeholders in anti-trafficking efforts, particularly in emergency care settings, and there is a need to strengthen their role, through training, standardized screening protocols, and institutional support, to more effectively support prevention, identification, and intervention efforts [12].

The Missing and Murdered Indigenous Women and Girls (MMIWG) movement has sparked attention to the recognition and awareness of modern-day trafficking of Indigenous women in Canada and the United States [6]. The movement has uncovered barriers created by social, political, media, and law enforcement influences that have hampered efforts to learn more about the impact of human trafficking on North American Indigenous women. For example, convoluted relationships between tribal and non-tribal law enforcement agencies provide unstable environments that contribute to the activity of human trafficking within Indigenous populations [6]. A gap exists among research, reported data, and actual prevalence of human trafficking of North American Indigenous women [6,14]. Furthermore, barriers in the healthcare systems of the United States and Canada limit Indigenous women who have been trafficked from accessing affordable, evidence-based, compassionate treatment for physical and mental health effects of human trafficking [3,6,15]. There is a need for a comprehensive evaluation of the available evidence about the human trafficking of North American Indigenous women to provide the best healthcare for the victims and survivors.

### Aims

Although human trafficking has been studied across various populations and countries, little research has focused on North American Indigenous women. This scoping review aimed to identify known risk factors for trafficking among North American Indigenous women and to synthesize the complexities of trafficking and its related health outcomes.

## 2. Materials and Methods

Evaluation of the available scientific literature on the human trafficking of North American Indigenous women was conducted with a scoping review to identify further concepts and gaps in research [16]. The review was guided by the following research questions: (1) what risk factors are associated with human trafficking among North American Indigenous women, and (2) what gaps exist in the current literature. Steps outlined in the PRISMA-ScR checklist were followed, including (1) providing a statement of the question and objectives, (2) stating eligibility criteria and information sources, (3) providing the electronic search strategy and process for selecting sources of evidence, (4) giving numbers of sources of evidence screened, (5) summarizing the main results, and (6) providing a general interpretation of the results [17]. This framework was utilized to synthesize knowledge about the trafficking of Indigenous women to support the advancement of nursing by informing evidence-based, culturally responsive care and guiding future research, education, and practice initiatives.

### 2.1. Data Sources

We conducted a systematic literature search with the assistance of a librarian. We searched the following databases, CINAHL, Embase, PubMed, SCOPUS, PsycINFO, Cochrane Library, and Google Scholar, in March 2022 and updated on 23 September 2023. Initial search terms were “human trafficking” paired with “North American Indians, “Native,” “Indigenous,” and “Indian.” We conducted another search using specific criteria of human trafficking, including “enslavement” (MeSH), “sex work” (MeSH), “sex trafficking,” “labor trafficking,” and “trafficking of women” paired with “North American Indians,” “Indian,” “Native,” “Indigenous,” and “tribe.” We conducted the third search using Google Scholar, and only the keywords “human trafficking,” “enslavement,” “sex work,” “sex trafficking,” “labor trafficking,” and “trafficking of women” paired with “Native American,” “Indian,” “Native,” “Indigenous,” “tribe” and “woman or female.” This search process is provided in Table 1.

### 2.2. Study Selection

**Figure 1:**
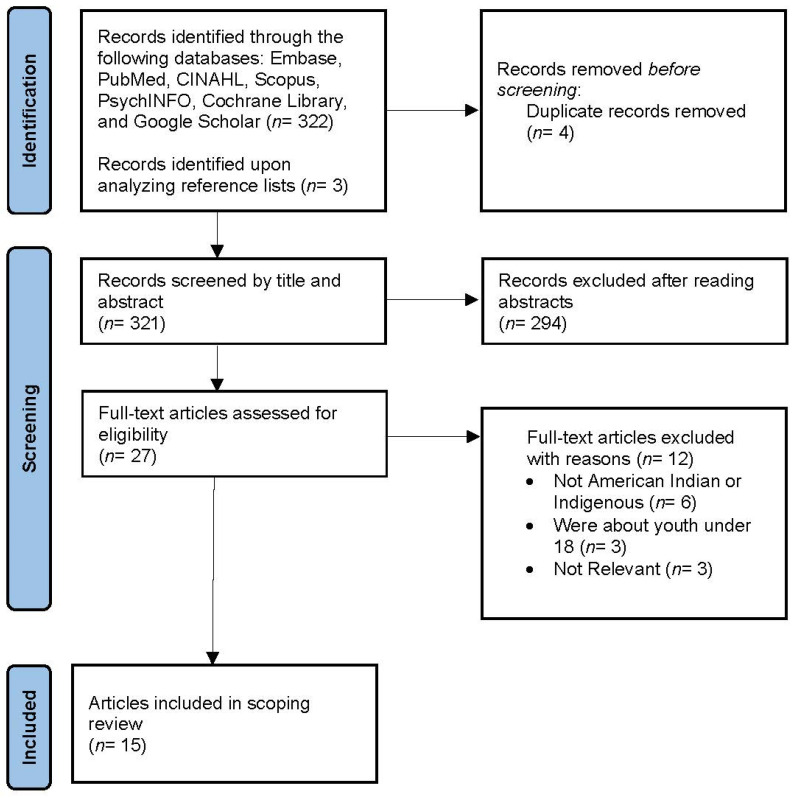
Flow Diagram of Article Selection [16].

The inclusion criteria for this scoping review were original research articles about human trafficking in Indigenous women from the United States and Canada as the primary source. Research reports, literature reviews, dissertations and theses, commentaries, and government reports were also included. Non-English articles, full-text unavailability, and articles that focused on populations under 18 years of age were excluded. Duplicates were removed using EndNote [Philadelphia, PA, USA], and the included articles were exported into an Excel [Redmond, WA, USA] spreadsheet for the title/abstract screening and full-text screening. The first two authors independently reviewed the articles and had 100% concordance at both screening stages. A third reviewer was identified had the two reviewers not agreed on including an article, but this reviewer was not utilized. The original database search yielded 322 articles; four articles were removed as duplicates. Throughout the writing process, we identified three new articles through reference combing. During the title abstract screening 294 articles were excluded as being non-relevant to the topic. Twenty-seven full-text articles were reviewed for eligibility, and 12 articles were excluded due to either not being within the identified North American Indigenous populations, being about children under age 18, or not being relevant to the research question (Figure 1).

### 2.3. Data Extraction and Synthesis

The first author extracted data from the articles in the form of a Word table with attention to the details of the population, the aim of the study or the literature, the key findings, and recommendations of the original authors. The second author corroborated the extracted data. The data were synthesized to identify key themes and presented in narrative format. The risk of bias in each article was not assessed, as this is not consistent with the purpose and methodology of a scoping review [17].

## 3. Results

### 3.1. Study Characteristics

The articles included in this scoping review came from the following databases: CINAHL (*n* = 1), Scopus (*n* = 5), Embase (*n* = 1), PsycINFO (*n* = 1), and Google Scholar (*n* = 7). Of the 15 articles, there were six original research articles consisting of qualitative studies (*n* = 2) [18,19], case studies (*n* = 2) [20,21], and mixed methods studies (*n* = 2) [22,23]. Other articles were from book chapters [24,25], a government report [26], a website [27], and literature reviews from peer-reviewed journals [28,29,30,31,32]. The literature represented the disciplines of criminology (*n* = 1) [31], gender-based violence (*n* = 1) [27], government (*n* = 1) [26], health (*n* = 1) [22], human trafficking studies (*n* = 2) [24,25], Indigenous research (*n* = 1) [32], law (*n* = 4) [23,28,29,30], nursing (*n* = 1) [20], social work (*n* = 2) [18,19], and urban planning (*n* = 1) [21]. These articles were from Canada (*n* = 3), Canada and the United States (*n* = 2), and the United States (*n* = 10). There were a total of 170 participants in the original research studies, with 14 participants in the qualitative studies, 5 participants in the case studies, and 151 participants in the mixed methods studies. Of those participants, 54 were professionals who worked with and supported Indigenous women. The other participants (*n* = 116) were Indigenous women whose ages ranged from 16 to 60 years and were victims of human trafficking.

### 3.2. Identified Themes

Among the 15 articles, we identified three themes: structurally produced vulnerabilities and other contributors to human trafficking, long-term effects of human trafficking, and collaboration of key stakeholders needed to end trafficking. Table 2 lists characteristics of the articles that were summarized.

#### 3.2.1. Structurally Produced Vulnerabilities and Other Contributors to Human Trafficking

The risk factors for Indigenous women to enter trafficking are discussed in all 15 articles and most frequently conceptualized as vulnerabilities. The most reported structural drivers of risk are shaped by broader social and structural conditions, and include personal factors, the historical and contemporary oppression of Indigenous communities, and inadequate systems to prevent and respond to trafficking.

Personal Factors: A personal or family history of sexual or physical abuse, substance use, or being in the child welfare system was consistently reported across studies, as were economic disparities and experiencing homelessness [22,23,24,26,27,30], all of which are often rooted in intergenerational trauma and structural inequities. Some Indigenous women trafficking survivors reported being lured into trafficking with the promise of a better life [21,23,24,30]. The physical and mental health sequelae of trafficking for women can perpetuate the trafficking due to paralyzing symptoms such as muscle pain, impaired memory, headaches, and post-traumatic stress disorder [22,24]. The geographic location of an Indigenous woman’s home or work can be a risk factor, as certain areas such as borders and ports are loci for human trafficking [21,25]. Importantly, North American Indigenous women experience disproportionately higher rates of trafficking and abuse by non-Indigenous men than by Indigenous men [21,24,30,31]. Bailey and Shayan (2016) determined that exposure to digital technologies such as the Internet has been a risk factor for the trafficking of Indigenous women victims and has enabled some perpetrators [28]. Trafficking survivors who identify as two-spirit or trans reported feeling isolated among their support services because they were not well understood [19,32]. Growing evidence demonstrates that vulnerability to human trafficking is produced through interconnected social and structural conditions, rather than solely individual-level factors.

Historical and Contemporary Oppression of Indigenous Communities: Colonization was described by the National Congress of American Indians Policy Research Center as a historical and contemporary risk factor for the trafficking of Indigenous women [7]. According to their description, before the appearance of Europeans on the continent in the 1600s and 1700s, Indigenous tribes had strong kinship systems and self-governance. Women were thought to be sacred and revered members of society. When the colonists came to the continent seeking religious and political freedom and economic opportunities, some of these men assumed the right to kidnap, assault, and prostitute the Indigenous women without consequence. The cultural assimilation policies that were enacted against Indigenous Peoples in North America, including boarding schools, forced relocation, and involuntary sterilization, augmented the violence towards Indigenous women. These traumatic historic acts have created cycles of systemic racism and oppression that persist today [7]. Seven articles in this review reported on the effects of racism and past and current effects of colonization that shape structural conditions increasing the risk of involvement in human trafficking [19,20,23,24,26,28,29]. Trafficking is an ongoing outcome of colonial systems and their contemporary manifestations.

Inadequate Systems to Prevent and Respond to Trafficking: Health prevention and treatment interventions for Indigenous women who have survived trafficking are rare [32]. The healthcare systems in Canada and United States largely fail to detect patients who are involved in trafficking and lack the holistic approach needed to address these women’s complex issues [20,22]. The criminal system is also blamed for inadequately addressing the human trafficking of Indigenous women [19,21,23,28]. Two articles discussed a lack of concern from law enforcement as they do not properly investigate human trafficking reports, discriminate against the women victims of human trafficking, or discredit the women or those who report the crimes [19,29]. Another example by Bourgeois (2015) described a legal loophole where Canada’s criminal code addresses physically violent means of trafficking but fails to prosecute traffickers who use means of deception, fraud or power to recruit victims [29]. Shanley and Jordan (2017) describe the challenges of prosecuting in the United States, where the jurisdictional authority depends on the type of crime, location, and Indigeneity of the victim and perpetrator [24]. Tribal, state, or federal courts sometimes fail to prosecute human traffickers (especially non-Indigenous perpetrators) [30], so the victims of trafficking are less likely to report their situation [24].

#### 3.2.2. Long-Term Effects of Human Trafficking

While human trafficking is an act towards an individual or individuals, the long-term effects of human trafficking not only impact the individual victim but also their families, communities, and healthcare providers [18,22,24,32]. Some of the Indigenous women trafficking survivors (*n* = 105) who participated in a mixed methods study reported experiencing repeated physical, sexual, mental, and verbal abuse during captivity that continued to affect them well after they left the trafficking situations. They described the abuse as coming from the “johns,” individuals paying for sex, the traffickers, and boyfriends [22]. Five articles described physical injuries of Indigenous women who have been involved in trafficking, including broken bones, jaw fractures, musculoskeletal injuries, dental and oral issues, pelvic injuries, back pain, traumatic brain injuries, weight loss, kidney infections, sexually transmitted infections, and damage to reproductive organs [19,22,24,32]. Furthermore, Indigenous women involved in trafficking are at higher risk of being threatened by a weapon or being a victim of a homicide [19,31].

Not all injuries that human trafficking survivors experience have physical manifestations, as other long-term effects include mental health issues from the trauma of the activity. Every article mentioned the emotional or mental health effects of human trafficking on Indigenous women. Four articles specified that survivors experience outcomes such as depression, anxiety, memory issues, suicidal ideations, post-traumatic stress disorder, difficulty concentrating, difficulty sleeping, chronic flashbacks, or post-trauma substance abuse issues [20,22,27,32]. Farley et al. (2016) interviewed 105 American Indian women in Minnesota who experienced trafficking, and they reported having frequent diagnoses of depression (78%), anxiety disorders (71%), and bipolar disorder (33%) [22]. While many of these physical and mental injuries have devastating impacts on the human trafficking victim, families and communities are impacted as well due to ongoing hospital, healthcare-related, and mental health costs [32].

#### 3.2.3. Collaboration of Key Stakeholders Needed to End Trafficking

Human trafficking involves complex socio-political issues and has become a severe public health crisis among women from North American Indigenous populations. Therefore, collaboration is needed to end trafficking. Every article in this scoping review identified this collaborative need and mentioned a range of stakeholders, from policymakers, law enforcement agencies, healthcare providers, social workers, communities, service providers, and educators to multidisciplinary researchers. Indigenous trafficking survivors who were interviewed about how they got out of trafficking reported a variety of systems, including hospitalization, family, and the police. The Indigenous researchers leading the study noted the irony that the same systems of healthcare, family and the police can sometimes be oppressive to Indigenous women [19]. Cumulative knowledge from these articles demonstrates that ending trafficking involves more than just implementing a new program. These efforts need to be holistic, culturally healing, and community-driven [19,22,32].

Collaboration of healthcare providers with individuals, families, communities, and professionals from other disciplines can inspire interventions such as that described by a Lakota nurse in North Dakota who serves Indigenous women trafficking survivors. Her focus is on holistic and uplifting recovery for women who have been trafficked, reminding each of their important contributions to the world [20]. A social work study found the importance of spirituality, ceremony, traditional healing, and person-centered care for the development of interventions for Indigenous women who have been trafficked [18]. Similarly, three other articles reported the need for attention to spiritual teachings and beliefs on the healing journey for Indigenous trafficking victims [19,22,27]. The needs of survivors include legal assistance, protection from abusers, housing, childcare, and trauma-informed job placement [21]. Victims and survivors also need equitable access to healthcare [19,24]. Social media may serve to help raise awareness about the human trafficking of Indigenous women and to improve access to justice for these women [28].

Finn et al. (2017) shared an approach to combatting sex trafficking on the Fort Berthold Reservation that incorporated engagement of relevant businesses, tribal capacity and coalition building, and remedies contained in the Violence Against Women Act of 2014 [30]. Another article described a statewide, multi-tier approach implemented a decade ago that has yielded improvements through three strategies: treating trafficking victims with care rather than arresting them for prostitution, targeting the demand for purchased sex, and prosecuting the traffickers themselves [24]. Collaborations between all tribal and non-tribal stakeholders are imperative to understanding, identifying, acknowledging, protecting, advocating for, and supporting victims (Table 2).

## 4. Discussion

This review aimed to determine what is known about the human trafficking of North American Indigenous women. This scoping review included six original research studies in which 116 Indigenous women who had been involved in trafficking and 54 service professionals who had worked with them shared their stories and experiences. The rich data from these studies provided a glimpse into the lived experiences of trafficking victims and survivors, though it is worth noting that women tend to underreport involvement in human trafficking due to shame, fear of retaliation, or distrust of law enforcement [24], challenges that also explain the paucity of research in this area. Although our search strategy was intentionally broad with no limits on publication date, only four of the fifteen articles were identified from healthcare and social work sources. This likely reflects differing disciplinary approaches to human trafficking. We do not interpret this as indicative of a hierarchy of evidence but rather it highlights the uneven representation of disciplinary perspectives within the accessible literature. Furthermore, the scant amount of original research available to guide evidence-based care of Indigenous women involved in trafficking suggests a need for all healthcare providers to familiarize themselves with this unique and tragic public health issue.

In this review, three common themes emerged: risk factors of trafficking as shaped by structural and colonial conditions, effects on women’s health, and the need for collaborative efforts to end human trafficking. When comparing findings of North American Indigenous human trafficking victims to women of other racial/ethnic groups, there are many similarities. Women trafficking survivors of all races and ethnic backgrounds were likely to have had a childhood with exposure to sexual or physical abuse, the child welfare system, substance use, economic disparities, or experiencing homelessness [7,14]. Similarly, the mental and physical sequelae of trafficking are similar across populations [5]. However, some Indigenous women trafficking survivors reported the compounded burdens of experiencing cumulative trauma from persistent systems of colonialism, complicated and ineffective tribal and non-tribal legal systems for reporting and prosecuting traffickers, and support resources that were not aligned with their cultural beliefs or traditions. Two areas warrant particular attention: historical trauma and culturally grounded healing practices. Smith (2012) urges that all research and work with Indigenous Peoples recognize the biases of Western historical and philosophical paradigms [33]. Only when researchers reflect on and critically examine their own beliefs can they possibly access Indigenous knowledge and other ways of knowing. Ultimately Indigenous-led research can inform culturally tailored prevention and treatment interventions for North American Indigenous women who have been involved in trafficking. Decolonizing research is aimed at benefiting the studied communities directly. A surprising result of this scoping review was how the data from articles from disciplines such as law, criminology, government, and urban planning intersected with the data from Indigenous studies and healthcare. This highlights the complex needs of Indigenous women involved in trafficking and emphasizes the need for a multidisciplinary call to action to prevent trafficking, support victims and help survivors on their healing journeys, as the potential healing available to North American Indigenous women is best achieved by implementing cultural methods and interventions [10]. There has been a recent spike in peer-reviewed research and commentary about decolonizing nursing coming from the United States and Canada [34]. For example, Rosario and colleagues [35] conducted a scoping review on nursing and decolonizing. They recommend that nurses working with North American Indigenous women in trafficking embrace the views and experiences of the women, creating pipelines for future Indigenous nurses to care for their communities, ensuring cultural safety in nursing practice and learning nursing’s historical role in colonization.

Furthermore, nurses are well-positioned, highly skilled, and knowledgeable to lead efforts to identify, protect and provide high-quality care for Indigenous women who are victims of violence [36]. Nurses have an opportunity to lead efforts to bring awareness and educate on this public health issue to combat human trafficking. These efforts include understanding the vulnerabilities of victims, recognizing the barriers to reporting, creating partnerships to support culturally appropriate care, and advocating for healthcare providers to implement and utilize available human trafficking screening tools for improved human trafficking victim identification [36]. Nurses can create culturally specific health programs to close gaps in health disparities between Indigenous populations and non-Indigenous populations. They can impact change within healthcare systems to provide more culturally appropriate services for those who have lived through human trafficking [34]. These actions will not only increase awareness and enhance education on human trafficking but can lead to improved health outcomes for North American Indigenous women.

### Strengths and Limitations

The strengths of this scoping review were the use of the PRISMA-ScR guidelines and inclusion of mixed-method, qualitative, and quantitative studies, literature reviews, and grey literature to increase knowledge about North American Indigenous women and human trafficking. Two authors (DT and CH) participated in independently screening and summarizing the articles and maintaining an audit trail. 

Limitations of this scoping review are acknowledged. First, only articles that explicitly stated human trafficking among North American Indigenous women were included, so other resources may have been missed, as there are a variety of definitions for the act of human trafficking. Second, although we included articles about LGBTG and two-spirit people, our search terms did not include “LGBTQ” or “two-spirit,” so important information may have missed about the trafficking of these populations by failing to include them in the search. Another limitation is the exclusion of studies involving populations under 18 years of age. While this approach allowed for a more focused examination of human trafficking experiences in the adult populations, it may have resulted in an incomplete understanding of the developmental pathways into trafficking (e.g., through foster care involvement, homelessness, or family violence) that often begins during adolescence. Finally, the unique values, beliefs, and traditions across each of the hundreds of Indigenous tribes in North America were not examined. It is important to note that Indigenous communities are not homogenous, and experiences are shaped by diverse cultural values, belief systems, traditions, and governance structures. The evidence about Indigenous women was generalized to gather as much evidence about trafficking as possible.

## 5. Conclusions

Human trafficking of Indigenous women is a serious public health issue. There are few research studies and limited general literature about the trafficking of Indigenous women. Many disciplines across healthcare lack an evidence base from which to create interventions to combat human trafficking, especially with women from cultures that experience historical trauma and contemporary systemic oppression. Trafficking, unfortunately, continues to go underreported, further complicating protection and support efforts. Despite the limited number of available studies, this scoping review summarized the vulnerabilities and risk factors contributing to the victimization of Indigenous women in human trafficking. Multiple sectors of healthcare, research, and government must address short- and long-term effects on the physical and mental health of Indigenous women when implementing collaborative efforts. Furthermore, these findings highlight the essential role of nurses to lead in developing holistic, culturally responsive interventions to provide protection and support to Indigenous women, their families, and their communities.

## Figures and Tables

**Table 1:** Search process.

Search Engines	Search Terms and Combinations	Search Results
CINAHL	(MH “Native Americans+”) OR TI ((“native american” or “american indian” or indigenous or “native tribe people”)) OR AB ((“native american” or “American indian” or indigenous tribes” or “native people”)) OR TI ((“human trafficking” or “sex trafficking” or enslavement)) OR AB ((“trafficking” or “sex trafficking” or enslavement)) AND (MH “human trafficking”)	All results (*n* = 10); after title/abstract (*n* = 3); final selection (*n* = 1)
Cochrane Library	“Human Trafficking”	All results (*n* = 1); after title/abstract (*n* = 0); final selection (*n* = 0)
Embase	‘American Indian’/exp OR ‘Native American’:ab, ti OR ‘American Indian’:ab, ti OR indigenous: ab, ti OR ‘native tribes’: ab, ti OR native: ab, ti AND ‘human trafficking’: ab, ti OR ‘sex trafficking’: ab, ti OR enslavement: ab, ti	All results (*n* = 22); after title/abstract (*n* = 1); final selection (*n* = 1)
Google Scholar	(“Human Trafficking” OR “enslavement OR “sex work” OR “human trafficking*” OR “sex trafficking*” OR “labor trafficking*” OR “trafficking of women”) AND ((“Native American” OR Indian OR native OR indigenous OR tribe OR tribal)) AND (woman or women or female)	All results (*n* = 15); after title/abstract (*n* = 8); final selection (*n* = 7)
PsycINFO	DE “Human Trafficking” OR DE “Sex Trafficking” OR TI ((“native american” or “American Indian” or indigenous or “native tribal people”)) OR AB ((“native American” or “American Indian” or indigenous tribes” or “native people”))	All results (*n* = 25); after title/abstract (*n* = 1); final selection (*n* = 1)
PubMed	(“Human Trafficking” [Mesh])) OR “human trafficking” [Title/Abstract] OR indigenous [Title/Abstract] OR Indian [Title/Abstract])) AND “Human Trafficking” [Mesh] AND Indian [Title/Abstract])) OR “Human Trafficking” [Mesh] AND indigenous [Title/Abstract] OR “Human Trafficking” [Title/Abstract]) AND native [Title/Abstract] OR “human trafficking” [Title/Abstract])) AND ((“Indians, North American” [Mesh])	All results (*n* = 12); after title/abstract (*n* = 0); final selection (*n* = 0)
Scopus	TITLE-ABS-KEY (“human trafficking” OR “sex trafficking” OR enslavement) AND (“native American” OR “American Indian” OR indigenous) AND (women OR woman OR female)	All results (*n* = 237); after title/abstract (*n* = 14); final selection (*n* = 5)

**Table 2:** Articles included in the scoping review.

Author (Year)	Literature Type or Methods and Source	Location and Population	Aims	Key Findings or Conclusions
Bailey & Shayan (2016) [28]	Review of Literature and Feminist Critique Law Journal	Canada First Peoples including First Nations, Inuit, and Métis	To consider how digital technologies are informed by, and implicated in, the missing and murdered Indigenous women crisis in Canada.	Digital communications technologies, such as the internet, are encoded to perpetuate power imbalances for women and First Peoples. Technologies interact with other socio-cultural and historical forces in ways that expose Indigenous women and girls to vulnerabilities that are recognized as the root causes of the missing and murdered Indigenous women crisis in Canada. Voluntary DNA programs are helpful in retribution, but do not address the root causes of trafficking. Digital communications should be involved in increasing victims’ access to justice and a national action plan.
Bourgeois (2015) [29]	Review of Literature Law Journal	Canada Indigenous women	To explore the role of the Canadian State in the human trafficking of Indigenous women and girls in Canada. A review of past and current litigation and an argument that Canada has been complicit in human trafficking of Indigenous women and girls in Canada.	While it is admirable the Canadian State has stepped up to fight human trafficking of Indigenous women, they remain complicit in the violence. To end human trafficking within the indigenous population, decolonization must take place, requiring a hard internal examination of the complexities of human trafficking.
Farley et al. (2016) [22]	Mixed-Methods Design Health Journal	Minnesota 81% Anishinaabe women	To examine social and physical violence against AI/AN women in prostitution and the impact on mental and physical health.	In total, 98% were currently or previously homeless. Common themes of vulnerabilities that led to human trafficking were homelessness, rape, assault, and racism. The victims had a high prevalence of physical and mental symptoms of muscle pain, impaired memory, headaches, and post-traumatic stress disorder. The women need collaborative approaches with healthcare, legal assistance, physical protection from a pimp, and childcare to overcome victimization.
Fear (2019) [20]	Case Study Nursing Journal	North Dakota Lakota women	To bring awareness of the problem of human trafficking in the Indigenous population. To describe the nursing care given to Native women victims of trafficking that empowers them and provides positive messaging.	This is a call to action: to truly know someone’s needs, healthcare professionals need to look past medication lists and reasons for being seen.
Finn et al. (2017) [30]	Review of Literature Law and Gender Journal	North Dakota Mandan, Hidatsa, and Airkara Nation women	To describe the intersection of sex trafficking and oil and gas development on the Fort Berthold Reservation; to describe jurisdiction and examine strategies to address the complex issue.	Native Americans are the only ethnic/racial group where missing person numbers are not tracked. Social risks are high with oil development in American Indian Reservations. Native American victims have overlapping risk factors that include exposure to domestic abuse, sexual assault, and poverty. Human trafficking will continue to be a silent crime unless all stake holders come together to combat the violence.
Hagen & Whittemore (2017) [26]	US Government Report	United States American Indian and Alaska Native Peoples	To outline federal jurisdiction from commercial sex trafficking offenses in Indian Country and to offer an example of the development of a comprehensive plan to combat human trafficking in Indian Country.	Collaboration is key, as working within American Indian reservations is complex due to multiple jurisdictions and a myriad of criminal justice and social services personnel. Risk factors include vulnerability, financial instability, and history of abuse and sexual assault. Improving public safety and the fair administration of justice in tribal communities is a top priority for the Department of Justice.
Hill, et al. (2022) [21]	Review of Literature and Case Studies Urban Environment Journal	Northwestern United States American Indian Women	To summarize the socio-political history contributing to risks of American Indian women, and to conceptualize the industry that profits from human trafficking as a “racial economy.” To empirically examine case studies of missing and murdered Indigenous women.	The legacy of colonialism contributes to the problem of trafficking of Indigenous women. American Indian women continue to be sexualized in images, which contributes to sexual demand. Homelessness and/or lack of stable relationships create vulnerability. Complicated legal and jurisdictional structures and socio-economic inequality lead to immobility and social exclusion from resources and contributes to human trafficking. Mobility needs to be considered in human trafficking prevention efforts.
Hintz (2016) [18]	Qualitative Social Work Thesis	United States Female professionals working with Native American Women in a Northern Plains Tribe	To determine the essential components of a culturally based healing intervention for Native American Women who have experienced being prostituted.	Six themes were identified: (a) spiritualty cannot be separated from therapeutic interventions, (b) ceremony as a diverse, integral component of healing, (c) the role of community in healing, d) therapeutic activities that connect to culture, (e) person-centered intervention planning and (f) the lack of recognition of traditional healing practices.
Monchalin, et al. (2019) [31]	Review of Literature Criminology Journal	United States and Canada Indigenous Peoples	To reinscribe the understandings of the racialization of homicide statistics, the tragedy of missing and murdered Indigenous women and girls.	The colonialist treatment of Indigenous Peoples is most evident with the missing and murdered Indigenous women crisis. Indigenous voices and experiences are invisible.
Olson-Pitawanakwat & Baskin (2021) [19]	Qualitative Feminist Social Work Journal	Toronto, Canada Social service providers and American Indian, Two-spirit, and transwomen victims/survivors of human trafficking from the Wendat, Anishinabek, and Haudenosunee Peoples	To examine the lived experiences of human trafficking of the participants.	Identified five themes: (a) pathways into human trafficking, (b) collective and intergenerational trauma caused by systems of colonization, (c) uniqueness of Two-Spirit/Queer/Trans experiences, (d) what helped victims get out, and (e) healing journeys.
Pierce, et al. (2011) [27]	Review of Literature Gender-based Violence Website	United States and Canada Indigenous women	To summarize published materials on the commercial sexual exploitation of Indigenous women and children in the U.S. and Canada.	Research is limited on human trafficking of Indigenous women and girls. Three main points drawn from existing research were: human trafficking is a critical problem in the U.S. and Canada, American Indian women are more heavily targeted in some regions, and traffickers prey on Indigenous women because of vulnerabilities.
Shanley & Jordan (2017) [24]	Book Chapter Human Trafficking	Minnesota and Oregon American Indian Peoples	To examine contemporary human trafficking in Indian Country.	Contributing factors to human trafficking include historical context, U.S. government policies, vulnerability, historical trauma, substance use disorders, mental health diagnoses, racism, poverty, homelessness, history of child welfare involvement, and jurisdiction loopholes. Examples of culturally relevant interventions to combat trafficking are shared.
Stark (2019) [25]	Book Chapter Human Trafficking	North America American Indian and First Nations Women	Examine the history of sex trafficking of Indigenous Women.	Disparities experienced by Native American and First Nations women make them more vulnerable to human trafficking. Geographic areas like borders and ports are loci. Collaboration is key to combating this issue, with a need to develop civil litigation and support services for human trafficking victims.
Stumblingbear-Riddle, et al. (2019) [32]	Review of Literature Indigenous Research Journal	United States American Indian/Alaska Native women, girls, and two-spirit people	To discuss the influence of gender/race/ ethnicity/Indigeneity on trauma and the outcomes of human trafficking.	Repeated exposure to trauma related to sexual and gender violence for AI/AN women and girls and two-spirit people leads to a focus on survival. Human trafficking has devastating effects on survivors, families, and communities. Advocates on the front lines who are combating human trafficking contribute to healing.
Weedn, et al. (2014) [23]	Mixed-Methods Law Journal	Oregon Native American Peoples who had prosecuting, protecting, or preventing roles	To measure whether federal, state, and local government officials are meeting their obligations under international, national, and state laws in prosecuting traffickers, protecting survivors, and preventing trafficking as it involves the Native American population in Oregon.	Themes: (a) officials are not meeting obligations to prevention, prosecution, and protection of American Indian human trafficking victims, (b) generational trauma is a major contributor to human trafficking, along with a relationship between foster care, homelessness, and vulnerability to human trafficking, (c) the underreporting of sex trafficking crimes within Native American communities, (d) the distrust of non-tribal law enforcement, (e) jurisdictional confusion, and f) lack of funding for traditional healing methods.

## Data Availability

No new data were created or analyzed in this study. Data sharing is not applicable.

## Public Involvement Statement

No public involvement in any aspect of this research.

## Guidelines and Standards Statement

This manuscript was drafted against the Preferred Reporting Items for Systematic reviews and meta-Analyses extension for Scoping Reviews (PRSMA-ScR) Checklist for scoping review research.

## Abbreviations

The following abbreviations are used in this manuscript:

AI/AN	American Indian/Alaska Native
LGBTQ	Lesbian, gay, bisexual, transgender, or queer
